# Genomic Analysis of *Wolbachia* from *Laodelphax striatellus* (Delphacidae, Hemiptera) Reveals Insights into Its “Jekyll and Hyde” Mode of Infection Pattern

**DOI:** 10.1093/gbe/evaa006

**Published:** 2020-01-20

**Authors:** Xiao-Li Bing, Dian-Shu Zhao, Jing-Tao Sun, Kai-Jun Zhang, Xiao-Yue Hong

**Affiliations:** Department of Entomology, Nanjing Agricultural University, Jiangsu, China

**Keywords:** *Wolbachia*, *Laodelphax striatellus*, cytoplasmic incompatibility, mutualistic, parasitic

## Abstract

*Wolbachia* is a widely distributed intracellular bacterial endosymbiont among invertebrates. The *w*StriCN, the *Wolbachia* strain that naturally infects an agricultural pest *Laodelphax striatellus*, has a “Jekyll and Hyde” mode of infection pattern with positive and negative effects: It not only kills many offspring by inducing cytoplasmic incompatibility (CI) but also significantly increases host fecundity. In this study, we assembled the draft genome of *w*StriCN and compared it with other *Wolbachia* genomes to look for clues to its Jekyll and Hyde characteristics. The assembled *w*StriCN draft genome is 1.79 Mb in size, which is the largest *Wolbachia* genome in supergroup B. Phylogenomic analysis showed that *w*StriCN is closest to *Wolbachia* from Asian citrus psyllid *Diaphorina citri*. These strains formed a monophylogentic clade within supergroup B. Compared with other *Wolbachia* genomes, *w*StriCN contains the most diverse insertion sequence families, the largest amount of prophage sequences, and the most ankyrin domain protein coding genes. The *w*StriCN genome encodes components of multiple secretion systems, including Types I, II, IV, VI, Sec, and Tac. We detected three pairs of homologs for CI factors CifA and CifB. These proteins harbor the catalytic domains responsible for CI phenotypes but are phylogenetically and structurally distinct from all known Cif proteins. The genome retains pathways for synthesizing biotin and riboflavin, which may explain the beneficial roles of *w*StriCN in its host planthoppers, which feed on nutrient-poor plant sap. Altogether, the genomic sequencing of *w*StriCN provides insight into understanding the phylogeny and biology of *Wolbachia*.

## Introduction

*Wolbachia* is a genus of Gram-negative intracellular endosymbiotic bacteria belonging to the family Rickettsiaceae (order Alphaproteobacteria). *Wolbachia* is one of the most widespread endosymbionts in animals, existing in about 40% of arthropod species (Zug and Hammerstein 2012), as well as some nematodes (Lefoulon et al. 2016). *Wolbachia* is the abbreviation for *Wolbachia pipientis* (Lo et al. 2007), which contains 16 supergroups (A–Q, except for G, which is a combination of A and B) (Baldo, Dunning Hotopp, et al. 2006; Lo et al. 2007; Haegeman et al. 2009; Ros et al. 2009; Augustinos et al. 2011; Bing et al. 2014; Glowska et al. 2015). In insects, the most common supergroups are A and B (Lo et al. 2007).

As a facultative endosymbiont in arthropods, *Wolbachia* is famous for manipulating host reproduction. *Wolbachia-*mediated reproductive manipulation includes producing infected females without males (parthenogenesis-inducing), feminizing genetic males (feminization-inducing), killing infected male progenies (male-killing), and inducing cytoplasmic incompatibility (CI) between infected males and uninfected (or differently infected) females (Werren et al. 2008). These phenotypes directly or indirectly result in a better survival environment for *Wolbachia*-infected females. In other words, they enhance *Wolbachia*’s maternal transmission and thus increase its ability to spread in populations. *Wolbachia* has also been shown to be beneficial in insects by protecting them from pathogenic viral infection and increasing host fitness and fecundity (Nikoh et al. 2014; Moriyama et al. 2015; Zug and Hammerstein 2015; Guo et al. 2018). In nematodes, *Wolbachia* functions as an obligatory mutualist endosymbiont (Comandatore et al. 2013).

The diverse effects of *Wolbachia* strains on their hosts are reflected in their genetic diversity. The diversity of genome features has elucidated insights of *Wolbachia*’s multiple functions in many insects, such as the mutualistic roles of *w*Cle (supergroup F, an obligatory *Wolbachia* from the bedbug *Cimex lectularius*) (Nikoh et al. 2014), CI induced by *w*Mel (supergroup A, *Wolbachia* from *Drosophila melanogaster*) (LePage et al. 2017), and male-killing from *w*Bol1 (supergroup B, *Wolbachia* from the butterfly *Hypolimnas bolina*) (Duplouy et al. 2013). Genomic analyses showed that *Wolbachia* strains have reduced genome size (Sinha et al. 2019), many pseudogenes, bacteriophages, and transposable mobile elements (Bordenstein and Wernegreen 2004; Wu et al. 2004; Gavotte et al. 2007; Klasson et al. 2009; Bordenstein and Bordenstein 2016), and genetic recombination events (Baldo, Bordenstein, et al. 2006; Zhang et al. 2013), which have contributed significantly to diversifying *Wolbachia* genomes.

The small brown planthopper *Laodelphax striatellus* (Fallén) (Hemiptera: Delphacidae) is one of the most destructive pests of rice *Oryza sativa* Japonica, the primary food source for half of the world’s population. *Laodelphaxstriatellus* feed on plant sap exclusively and lay eggs in plant tissues, causing direct physiological damage to crops. In addition, *L. striatellus* can migrate long distances and transmit many rice virus diseases (such as rice stripe virus) (Nault 1994). Previous field investigations have shown that *L. striatellus* is highly infected by *Wolbachia* (more than 90%) (Hoshizaki and Shimada 1995; Zhang et al. 2013). Microbiota analysis of field planthoppers showed that *Wolbachia* accounts for more than 80% of the identified operational taxonomic units (OTUs), indicating its dominance in *L. striatellus* (Bing et al., 2020, under revision). *Wolbachia* has been reported to increase host fecundity (Guo et al. 2018) and induce strong CI (Noda 1984a, 1984b) in *L. striatellus*, a classic type of “Jekyll and Hyde” mode of infection pattern (Jiggins and Hurst 2011; Moriyama et al. 2015; Zug and Hammerstein 2015). In addition, *Wolbachia* isolated from *L. striatellus* was recently shown to block the growth of several positive-sense RNA viruses (Dengue virus, Chikungunya virus, Zika virus, and yellow fever virus) by more than 99.9% in mosquito *Aedes albopictus* cells (Schultz et al. 2018), which increased its potential to block arbovirus infections in vectors. By having both mutualistic and deleterious effects in the same host species, the *Wolbachia* strain in *L. striatellus* is a good model for exploring the mechanisms of *Wolbachia*–host interactions.

We recently analyzed the genome sequence of *Wolbachia* in *L. striatellus* (*w*StriCN) to understand the molecular mechanisms underlying its beneficial effects (Ju et al. 2019). In this article, we provide a more detailed description of the *w*StriCN genome, including how it differs from other *Wolbachia* genomes, and unique features that may explain *Wolbachia*’s expansion, the separation of *Wolbachia* into supergroups A and B, the functions of *Wolbachia* in insect hosts, and how *Wolbachia* and the host communicate.

## Materials and Methods

### Insect Preparation

*Laodelphax striatellus* planthoppers were collected at a rice farm field in Nanjing, China, in July 2014 and then were maintained under laboratory conditions on rice seedlings in a climate-controlled room (25 °C, 60% RH, and an 8-h photoperiod). The *Wolbachia* infection statuses of individual *L. striatellus* were checked with polymerase chain reaction (PCR) and were confirmed with Sanger sequencing as previously described before (Zhang et al. 2013).

### *Wolbachia* Genome Sequencing, Assembly, and Annotation

The process of *Wolbachia* genome sequencing and assembly has been described in detail in Ju et al. (2019). The genome size and GC content of assembled *Wolbachia* genome were calculated with bioawk (https://github.com/lh3/bioawk/, last accessed January 19, 2020). The structural and functional annotation was predicted with Prokka 1.13.3 (Seemann 2014). The insertion sequences (IS) were identified based on ISfinder database (Siguier et al. 2006). Prophage sequences (WO) were annotated with PHASTER (Arndt et al. 2016). *Wolbachia* genomes were functionally characterized and compared by assigning predicted proteins to the following databases: Clusters of Orthologous Groups (COG) of proteins (Galperin et al. 2015), KEGG Ortholog database (Kanehisa and Goto 2000), and eggNOG (Huerta-Cepas et al. 2016). The signal peptides and transmembrane domains of proteins were identified with SignalP 4.0 (Petersen et al. 2011) and TMHMM 2.0 (Krogh et al. 2001), respectively.

### Genome Completeness Assessment

The completenesses of *Wolbachia* genomes were estimated with the BUSCO pipeline 3.0.2 (Waterhouse et al. 2018), which was performed against 221 highly conserved, single-copy orthologs (BUSCO groups) obtained from 1,520 proteobacterial species (proteobacteria_odb9) (Loetscher et al. 2016). To compare genomic features of various *Wolbachia* strains, all 53 available insect-associated *Wolbachia* genomes (by the time of April, 2019, supplementary table S2, Supplementary Material online) were downloaded from NCBI RefSeq or GenBank assembly database (Benson et al. 2018; Marchler-Bauer et al. 2018). Nematode-associated *Wolbachia* were excluded from this study because they differ distinctly in biology and phylogeny. As a low BUSCO score may indicate a poorly assembled *Wolbachia* genome and the complete *Wolbachia* genomes have BUSCO score >80% (Lindsey et al. 2016; Sinha et al. 2019), we used a complete BUSCO score of 80% as the criterion for genome quality control. The *w*StriCN and 32 other genomes were selected for further comparative genomic analyses. The 33 genomes were annotated all with the same methods mentioned above. *w*StriCN-specific genes were predicted with OrthoFinder version 2.2.6 (Emms and Kelly 2015). Synteny between *w*StriCN and other *Wolbachia* genomes was analyzed with NUCmer and visualized with mummerplot on Mumer 4.0.0beta2 (Kurtz et al. 2004). Average nucleotide identities between *Wolbachia* genomes were calculated using OrthoANI 1.40 (Yoon et al. 2017).

### Phylogenetic and Phylogenomic Analyses

For molecular phylogenetic analysis, we inferred *Wolbachia* phylogeny using 52 ribosomal protein sequences (twenty-one 30S ribosomal proteins and thirty-one 50S ribosomal proteins), and the *Wolbachia* ortholog groups generated by OrthoFinder 2.2.6 (Emms and Kelly 2015). The protein sequences were concatenated with SeqKit 0.9.2 (Shen et al. 2016), aligned with MAFFT 7.402 (Katoh and Standley 2013), and trimmed with Gblocks 0.91b (Castresana 2000). The best-fitting nucleotide substitution model was calculated with ModelFinder (Kalyaanamoorthy et al. 2017). The maximum likelihood (ML) phylogenetic tree was constructed with IQ-TREE 1.6.5 (Nguyen et al. 2015). The node support was calculated with 1,000 ultrafast bootstraps. The phylogenetic tree was visualized with ggtree (Yu et al. 2017) and was annotated in Inkscape (https://inkscape.org/).

### Analysis of Genome Content among *Wolbachia* Strains

COG category annotations were used to compare genome contents across the 33 *Wolbachia* strains. The number of genes in each category for each genome was subjected to principle component analysis (PCA) using R 3.5 (R Core Team 2018). The Wilcoxon rank-sum test was used to test for a significant difference in abundance of genes between supergroups A and B.

### Identification of Cif Proteins in *w*StriCN

Candidate CifA and CifB proteins in *w*StriCN were identified by searching similar proteins of known Cif proteins with Blast 2.7.1+ (Camacho et al. 2009) and OrthoFinder 2.2.6 (Emms and Kelly 2015). The phylogeny of Cif protein sequences was constructed as described above. Cif protein structures were predicted with HHpred (Söding et al. 2005) following Lindsey’s method (Lindsey et al. 2018).

## Results

### Genome Assemblies and Annotation of *w*StriCN

To obtain the genome of *w*StriCN, we generated two Illumina paired-end libraries (one with PCR and one without PCR) and one mate pair library on the Illumina HiSeq2000 platform. These libraries yielded 210-Mb (117× coverage of assembled *Wolbachia*), 211-Mb (118× coverage), and 457.6-Mb (280× coverage) data, respectively. In addition, we also generated a 454-pyrosequencing library that yielded 385.3-Mb (216× coverage) data on the Roche 454 GS FLX Titanium platform (supplementary table S1, Supplementary Material online). The draft genome of *w*StriCN was assembled with de novo assembly of Illumina reads with SOAPdenovo (Li et al. 2010) and closing gaps with 454 reads and Sanger sequencing results. The assembled *w*StriCN draft genome contains two scaffolds, composed of 42 contigs. The N50 of the contigs was 114,888 bp, about 142 times the average gene length (table 1). The total length of the *w*StriCN genome is 1,786,382 bp, which is one of the largest insect-associated *Wolbachia* genomes so far (table 1). The average GC content of the *w*StriCN genome is 33.72%, which is within the typical range of *Wolbachia* genomes (31.7% ∼ 38.3%) (supplementary table S2, Supplementary Material online).

**Table 1.** Genome Statistics of *w*StriCN

	*w*StriCN
Host	*Laodelphax striatellus*
Phenotypes	Cytoplasmic incompatibility, increase fecundity
Number of scaffolds	2
Number of contigs	42
Total nucleotides	1,786,382
N50 scaffolds	1,600,254
N50 contigs	114,888
GC content (%)	33.72
Number of CDS	1,747
Number of tRNAs	34
Number of rRNAs	3
Number of tmRNA	1
Average gene length	808.7

The Prokka pipeline annotation results (Seemann 2014) revealed that the *w*StriCN genome contained 1,747 protein coding sequences (CDSs) (92.8% of all *w*StriCN genes), 34 tRNA genes that could transfer all 20 amino acids, 3 rRNA genes (5S, 16S, and 23S rRNA), and 1 tmRNA gene (table 1). The 1,747 CDS-encoded proteins encompassed 182 complete and single-copy, 1 complete and duplicated, 5 fragmented and 33 missing highly conserved, Benchmarking Universal Single-Copy Orthologs (BUSCO groups), resulting in 82.81% BUSCO completeness score (supplementary fig. S1, Supplementary Material online). That score falls within the range of scores for published complete *Wolbachia* genomes (supplementary table S2, Supplementary Material online) (Sinha et al. 2019), which indicates the *w*StriCN genome is sufficiently reliable for further comparative genomic analyses.

### Taxonomy and Synteny of *w*StriCN

**Fig. 1.**
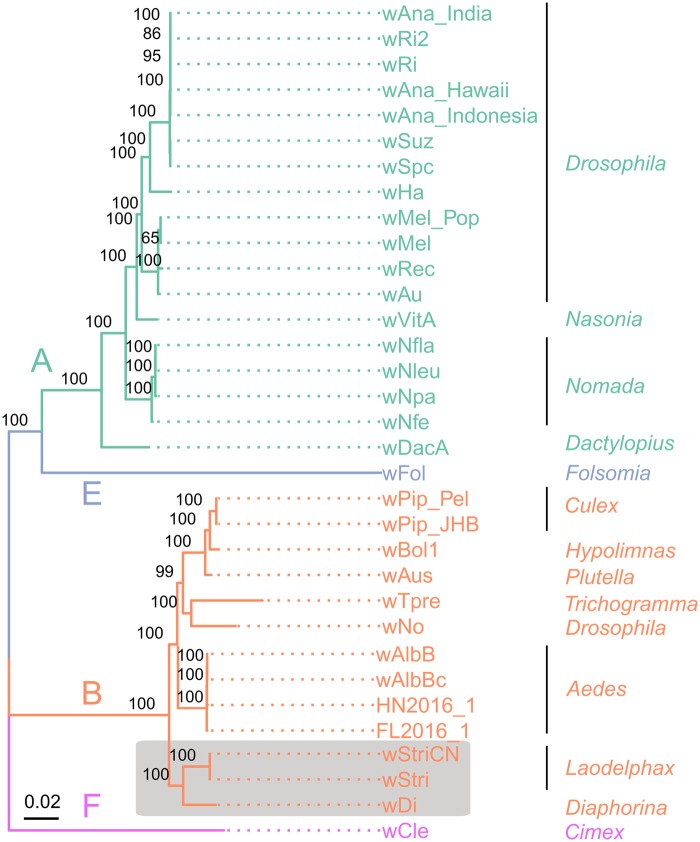
—Phylogenetic relationship of *Wolbachia* strains. The ML tree was calculated with a concatenated protein sequence of 367 single-copy protein sequences (215,584 amino acids) using an HIVw + F + R8 substitution model. *Wolbachia* supergroups are color coded as shown on the branches. The host genera of the *Wolbachia* strains are shown on the side. Bootstrap values are indicated at the respective node (only values >50% are shown). The scale bar represents the average number of substitutions per site.

It has been argued that the classic multilocus sequence typing (MLST) loci for *Wolbachia* (Baldo, Dunning Hotopp, et al. 2006) are problematic and may not reflect the properties of a *Wolbachia* strain very well (Bleidorn and Gerth 2017). Therefore, we performed two genome-wide phylogenomic analysis using 52 ribosomal protein coding genes (22,386 bp) and 367 single-copy orthologs of insect-associated *Wolbachia* genomes (215,584 aa), respectively. Both phylogenetic trees allocated *w*StriCN to supergroup B (fig. 1 and supplementary fig. S2, Supplementary Material online). In addition, *w*StriCN, wStri (the *Wolbachia* from a Korean *L. striatellus* population), and *w*Di (the *Wolbachia* isolated from the Asian citrus psyllid *Diaphorina citri*), clustered together and formed a strongly supported monophyletic group that was distinct from the other supergroup B *Wolbachia* members (fig. 1), suggesting they are close relatives. The average nucleotide identity (ANI) between *w*StriCN and *w*Stri/*w*Di genomes is 97.96%, which is much higher than the ANI between *w*StriCN and the other *Wolbachia* (88.88%) (supplementary table S3, Supplementary Material online). The above results suggest that these *Wolbachia* strains shared the same ancestor.

The colinear regions of MUMmer dot plots between pairs of *w*StriCN and other supergroup B *Wolbachia* genomes are larger than those between pairs of *Wolbachia* genomes of other supergroups (supplementary fig. S3, Supplementary Material online). In agreement with the finding of a high level of genome rearrangement between multiple *Wolbachia* genomes (Klasson et al. 2008; Duplouy et al. 2013), many inversions were found between *w*StriCN and other *Wolbachia* genomes (supplementary fig. S3, Supplementary Material online). It is noteworthy that the *w*StriCN sequences are more colinear with draft genomes than complete genomes of supergroup B *Wolbachia*. That may be because the directions of many scaffolds were artificially rearranged during the Mummer analysis. Therefore, it is necessary to be cautious when comparing rearrangements between draft genomes.

### Insertion Sequences

*Wolbachia* associated with arthropods are known to carry a large number of mobile elements, such as IS and WO (Kent and Bordenstein 2010; Bouchon et al. 2011), which may explain the expansion in *Wolbachia* genomes. As the largest genome in B supergroup so far, *w*StriCN genome was expected to be enriched in these features.

**Fig. 2.**
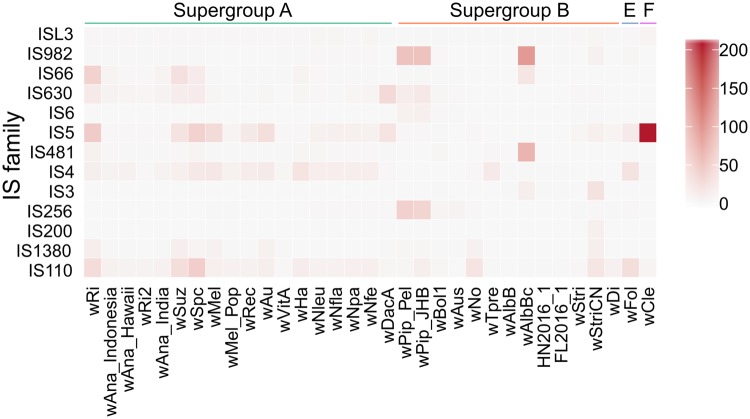
—Comparison of IS families from different *Wolbachia* supergroups. Abundance of IS elements in different IS families in different *Wolbachia* supergroups.

The ISfinder database identified 78 IS elements of 10 IS families from the *w*StriCN genome (supplementary table S5, Supplementary Material online). The most abundant IS families in *w*StriCN were the IS3 and IS110 families, containing 21 and 18 genes respectively. The total size of IS elements is 64224 bp, accounting for 3.6% of the *w*StriCN genome, which is much smaller than the IS elements in *Wolbachia w*Fol and *w*Pip (Klasson et al. 2008; Kampfraath et al. 2019). The top three IS families in abundance in all 33 invertebrate-associated *Wolbachia* genomes are IS5, IS110, and IS982 (supplementary table S5, Supplementary Material online). The *w*Cle genome harbors the highest number of IS elements, 96% (208/217) of which belong to IS5 (supplementary table S5, Supplementary Material online). Significant differences were found in the distribution of IS families among various *Wolbachia* genomes (fig. 2, chi-square test, *χ*^2^ = 3481.7, df = 384, *P* value < 0.0001). Within the same *Wolbachia* supergroup, the numbers of IS elements in different IS families also differ substantially (fig. 2). This is particularly striking for the *w*AlbB and *w*Tpre genomes, which are all supergroup B members (Lindsey et al. 2016; Sinha et al. 2019). It should be noted that the number of IS elements may be underestimated for *Wolbachia* genomes that are incomplete.

### Bacteriophage Genes

The PHASTER server predicted that the *w*StriCN genome has nine prophage regions (WOStriCN1-9), with a combined size of 233.9 kb (supplementary table S5, Supplementary Material online). These regions account for 13.1% of the *w*StriCN genome, which is a major contributing factor to the large genome size of *w*StriCN. Blasting prophage region sequences (WOStriCNs) against COG database revealed they encode modules for essential functions such as head or tail formation. For instance, WOStriCN5 contains genes encoding capsid proteins, tail formation proteins, tail sheath proteins, tail tube proteins, baseplate assembly protein, and phage terminase (supplementary fig. S4, Supplementary Material online). In addition, modules for the assembly of baseplate (wStriCN_00262, wStriCN_00303) and tail (wStriCN_00255, wStriCN_00299) are homologous to the P2 phage modules. The regions between the phage modules are mostly IS elements (transposases and other related proteins), ankyrin repeat (ANK) containing proteins and genes of unknown function (supplementary fig. S4, Supplementary Material online). In the WO regions of the *w*StriCN genome, the most significantly enriched elements other than phage-related genes were IS elements and ANK genes. Seventeen of the 78 IS elements in the genome and 32 of the 113 ANK genes in the genome were in the WO regions, indicating significant enrichment in the WO regions (binomial tests, *P* = 0.029 and <0.0001, respectively). Other genes that were significantly enriched in WO regions include site-specific DNA recombinase genes (10 out of 13, binomial test, *P* value < 0.0001), periplasmic serine protease of ClpP class (7 out of 9, binomial test, *P* value < 0.0001), and proteins with Zn-binding Pro-Ala-Ala-Arg (PAAR) domain, which were involved in Type VI secretion (8 of 8, binomial test, *P* value < 0.0001). The latter genes may have been horizontally shuttered by phage from *Wolbachia*. The closest matches of these genes in the NCBI NR databases were other *Wolbachia* genes.

### Functional Categories of *w*StriCN Genes

The representation of functional categories in *Wolbachia* genomes was analyzed by assigning the CDSs to COGs and KEGG databases. Of the 1,747 CDS in *w*StriCN genome, 1,339 CDSs (76.6%) were annotated to COG and 722 CDSs (41.3%) were annotated to KEGG pathways by the KAAS annotation server. KEGG analysis showed wStriCN contained complete pathways for the tricarboxylic acid cycle (map0020), fatty acid biosynthesis (map0061), oxidative phosphorylation (map00190), and lipoic acid metabolism (map00785), which are for essential energy metabolism. It also contained genes for biotin synthesis (map00780), riboflavin synthesis (map00740), and peptidoglycan biosynthesis (map00550). COG analysis showed that mobilome-related genes, such as prophages and transposons (category X, *n *=* *307), were the most abundant genes in *w*StriCN, in agreement with the large number of WO and IS elements described above. In the 33 *Wolbachia* genomes, the COG categories with the most variation in gene number per genome were mobilome, prophages, and transposons (X) and signal transduction mechanisms (T). The categories that showed little variance were secondary metabolites biosynthesis, transport and catabolism (Q), cell motility (N), and inorganic ion transport and metabolism (P) (supplementary table S6, Supplementary Material online).

**Fig. 3.**
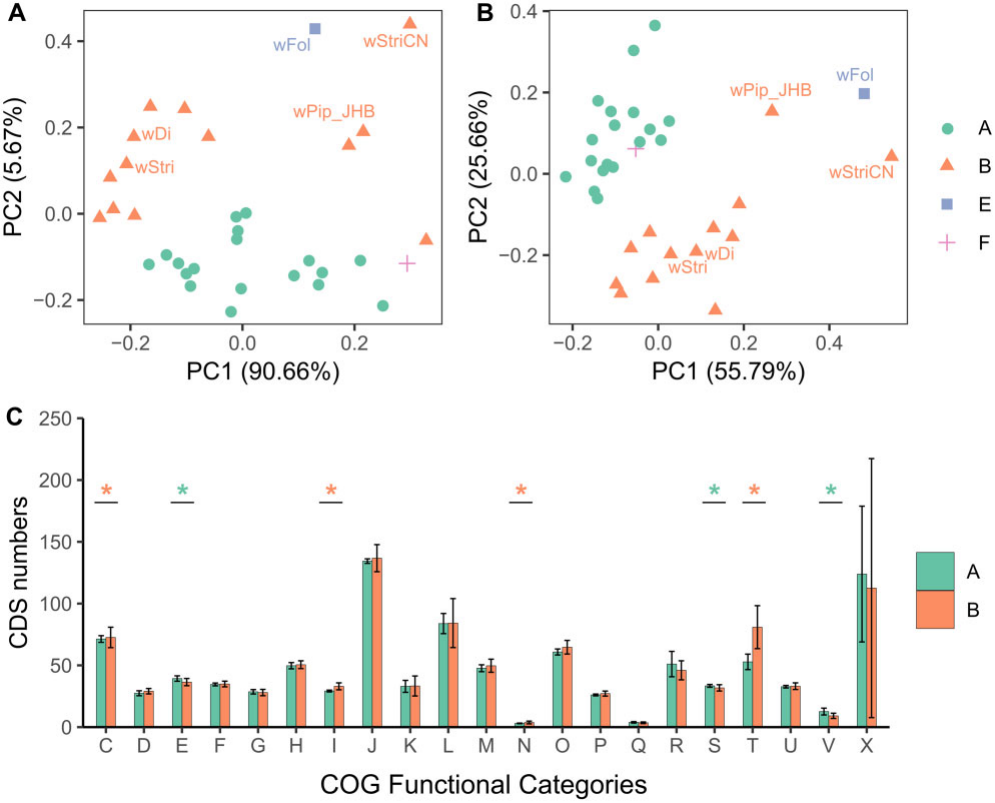
—Comparison of COG categories from different *Wolbachia* supergroups. (*A*) PCA of *Wolbachia* genomes based on the proportion of annotated genes in each COG category. (*B*) PCA of *Wolbachia* genomes excluding X category genes. (*C*) Bar chart comparing COG categories between *Wolbachia* supergroups A and B. Abbreviations of COG categories are C, energy production and conversion; D, cell cycle control, cell division, and chromosome partitioning; E, amino acid transport and metabolism; F, nucleotide transport and metabolism; G, carbohydrate transport and metabolism; H, coenzyme transport and metabolism; I, lipid transport and metabolism; J, translation, ribosomal structure, and biogenesis; K, transcription; L, replication, recombination, and repair; M, cell wall/membrane/envelope biogenesis; N, cell motility; O, posttranslational modification, protein turnover, and chaperones; P, inorganic ion transport and metabolism; Q, secondary metabolites biosynthesis, transport, and catabolism; R, general function prediction only; S, function unknown; T, signal transduction mechanisms; U, intracellular trafficking, secretion, and vesicular transport; V, defense mechanisms; X, mobilome, prophages, and transposons.

PCA based on the proportion of genes in each of the COG categories showed that supergroups A and B clustered separately by PC2. The factor that contributing to the PC1 (90.66% of total variance) and PC2 (5.67% of total variance) was the mobilome category which includes mainly prophages and transposons, and signal transduction mechanisms. The signal transduction category mainly consists of ANKs (fig. 3*A*). PCA without the mobilome category did not change the clustering of supergroups A and B (fig. 3*B*). *w*Cle clustered with supergroup A *Wolbachia*, and *w*Fol (the *Wolbachia* infecting springtail *Folsomia candida*) clustered on the side of supergroup B *Wolbachia* (fig. 3*A*), although they are phylogenetically distinct from each other (fig. 1). *w*StriCN’s closest neighbor in the PCA figure was *w*Fol, which may be because of the high number of mobilome genes and ANKs in both genomes (supplementary table S6, Supplementary Material online).

Additionally, the number of genes belonging to clusters in COG categories of amino acid transport and metabolism (E), defense mechanisms (V), and function unknown (S) were significantly more in supergroup A *Wolbachia* than that of supergroup B (Wilcoxon rank-sum test, *P* value < 0.05). The genes in COG categories related to energy production (C), lipid transport and metabolism (L), cell mobility (N), and signal transduction (T) were much more in supergroup B *Wolbachia* than supergroup A (Wilcoxon rank-sum test, *P* value < 0.05) (fig. 3*C*). Above data indicate distinct evolutionary pattern of *Wolbachia* in the two supergroups.

Ortholog analysis of Prokka-predicted proteins from the 33 *Wolbachia* genomes yielded 367 single-copy orthologs, from which 346 genes were assigned to COG database. Category analysis showed that half of the annotated genes were related to five COG categories: biological processes of translation, ribosomal structure and biogenesis (J), energy production and conversion (C), posttranslational modification (O), protein turnover, chaperones, replication, recombination and repair (L), and nucleotide transport and metabolism (F) (supplementary fig. S5, Supplementary Material online). These pathways are usually conserved and are usually involved in basic biological processes of an organism.

### Secreted and Transmembrane Proteins in *w*StriCN

SignalP and TMHMM predicted that the *w*StriCN genome has 33 proteins harboring signal peptide and 326 proteins with transmembrane helices. The secreted proteins were mainly proteins involved in forming the outer membrane, basic metabolite transportation, energy production, and secreted systems. The genome also contains proteins that may be associated with bacterial infection, such as the Invasion-associated locus B (IalB) (wStriCN_00836), a peptidoglycan hydrolase Rare lipoprotein A (RlpA) (wStriCN_00335), a Peptidoglycan deacetylase (PgdA) (wStriCN_00552), and the Outer membrane protein OmpA (wStriCN_00807). The proteins with predicted transmembrane-domains included mostly ANKs (37), energy production and conversion related proteins (34), proteins of secretion systems (20), inorganic ion transport related proteins (18), and phage tail proteins (12).

**Fig. 4.**
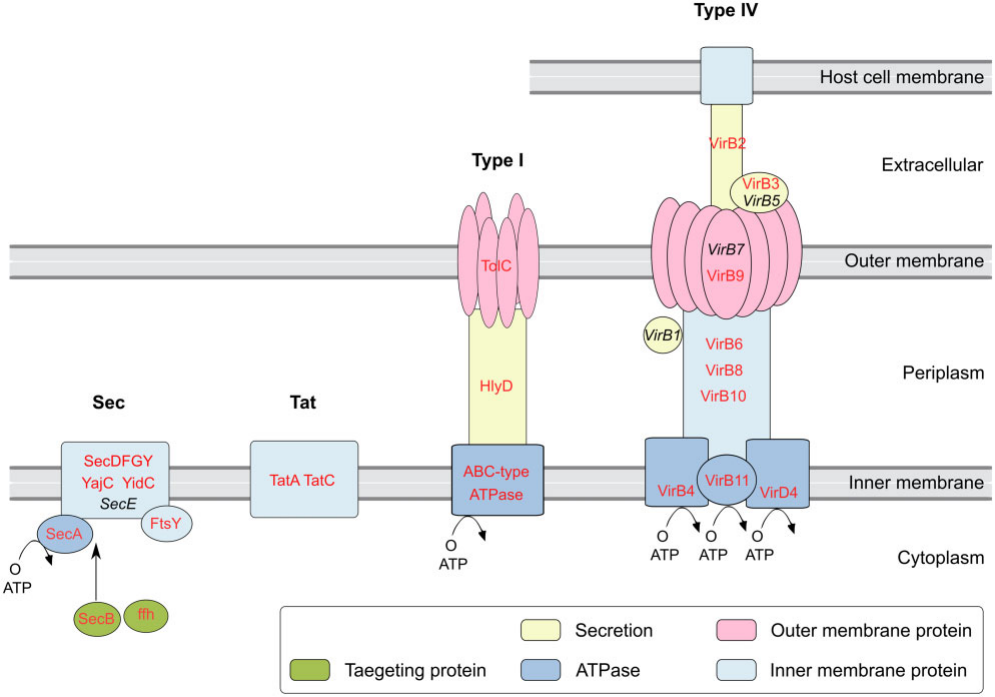
—Schematic view of secretion systems identified in the *w*StriCN genome. The figure is based on KEGG pathway map 03070. Proteins detected in wStriCN are colored red and those missing from *w*StriCN are shown in italics and black. The GspD from T2SS and PAAR proteins from T6SS were excluded, as they were too few to assemble the secretion systems.

*w*StriCN has various secretion pathways including the Type I secretion system (T1SS), Type II secretion system (T2SS), Type IV secretion system (T4SS), Type VI secretion system (T6SS), Sec secretion system, and Tat secretion system. Several proteins related to T1SS, the ABC-type protease ATPase (wStriCN_00721), outer membrane protein TolC (wStriCN_00600), and the fusion protein HlyD (wStriCN_01572) were detected in *w*StriCN (fig. 4). T1SS bypasses the periplasm and allows the secretion of proteins of diverse sizes (Delepelaire 2004). For instance, T1SS has been reported to secrete some ANKs in *Wolbachia*’s relative *Rickettsia* (Kaur et al. 2012). *w*StriCN contains one *GspD*/*PulD* gene (wStriCN_00409), which may create a pore in the outer membrane of the bacterial cell through which proteins can be secreted in the Type II secretion system (Nivaskumar and Francetic 2014). The *GspD*/*PulD* gene was possibly obtained by horizontal transfer as it is the only T2SS gene in *w*StriCN genome.

The *w*StriCN genome has a T4SS with 17 genes organized in 2 operons and 6 individual genes (fig. 4). One operon contains 4 gene copies of *virB6*, one *virB4*, and one *virB3* (wStriCN_00590 - wStriCN_00595). The other operon contains *virB8*, *virB9*, *virB10*, *virB11*, and *virD4* (wStriCN_01296 - wStriCN_01300). Three duplicated genes: *virB4* (wStriCN_00628), *virB9* (wStriCN_01017), and *virB8* (wStriCN_01520) are scattered in other parts of the genome. The gene organization of the T4SS is the same as it is in most other *Wolbachia* strains (Pichon et al. 2009). In addition, *virB2* has three homologs (wStriCN_00829, wStriCN_01226, and wStriCN_01313) in *w*StriCN.

We also identified eight genes harboring Zn-binding PAAR domain (supplementary table S8, Supplementary Material online), which are reported to be essential for T6SS-mediated secretion. Six genes forming the Sec secretion pathway (*secA, secB, secD, secF, secY*, and *secG*) and one *tatC* (wStriCN_01495) and two copies of *tatA* (wStriCN_00457 and wStriCN_01583) related to Tat translocases in the twin arginine translocation (Tat) pathway were identified as well (fig. 4). The Sec and Tat pathways are most commonly used in bacterial secretion systems to transport proteins across the cytoplasmic membrane. The Sec pathway primarily translocates proteins in their unfolded state, whereas the Tat pathway primarily secretes folded proteins (Green and Mecsas 2016).

### CI Genes in *w*StriCN

**Fig. 5.**
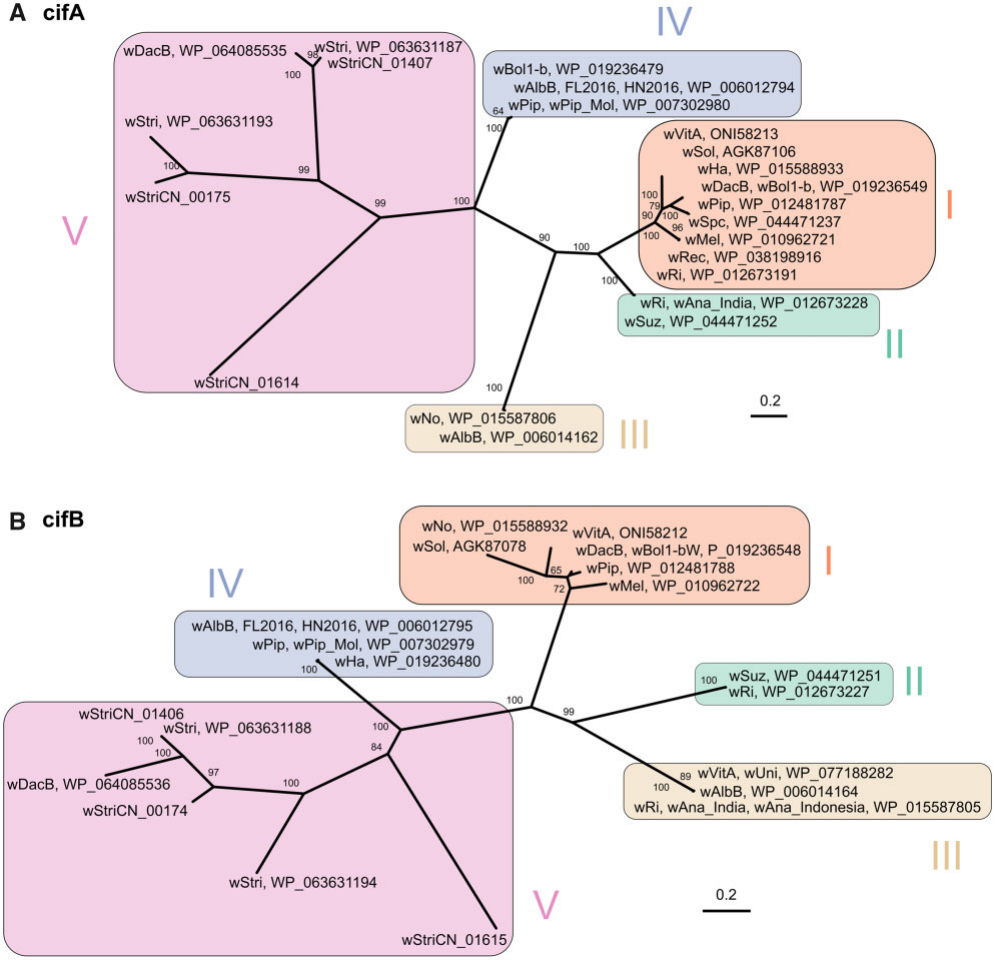
—Phylogeny of CifA (*A*) and CifB (*B*) proteins. The names of *Wolbachia* strains and the corresponding NCBI accession numbers of Cif proteins are shown. The tree was constructed using a JTT + F + G4 substitution model for ML analysis. Bootstrap values are indicated at the respective node (only values >50% are shown). The scale bar represents the average number of substitutions per site.

*w*StriCN has three tandem pairs of *cifA–cifB* genes (fig. 5). Phylogenetic analysis of CifA and CifB protein sequences showed they were all distinct from the four previously identified “Types” (Lindsey et al. 2018) and were assigned as Type V. The pair of wStriCN_01406 and wStriCN_01407 had similar homologs in *w*Stri and *w*DacB. The pair of wStriCN_00174 and wStriCN_00175 were only closely related in *w*Stri. The third pair, wStriCN_01614 and wStriCN_01615, were phylogenetically monophyletic (fig. 5). Altogether, CifA and CifB are abundant in *w*StriCN and have been diverged for a while.

**Fig. 6.**
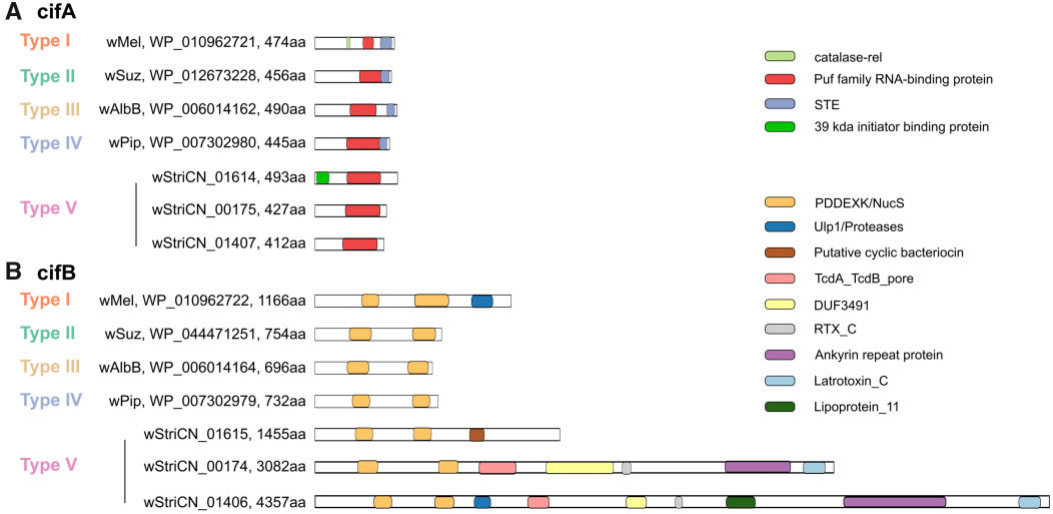
—Representative predicted structures of CifA (*A*) and CifB (*B*) proteins. Representative structures are shown for each type, with the *Wolbachia* strain name, accession number, and length of the protein indicated at the N-terminus. The structures of all three sets of Cif proteins from *w*StriCN are also shown.

The CifA proteins in *w*StriCN have two modules: a 39-kDa initiator inhibitor binding domain, which is a new module and locates only in wStriCN_01614, and a Puf family RNA-binding domain that locates in all three CifA homologs and other types of known CifA (fig. 6*A*). The STE-like transcription factor domain that was present in all other four types was not found in wStriCN CifA proteins. All *w*StriCN CifB proteins were predicted to have two PDDEXK/endonuclease NucS domains (fig. 6*A*), which are the same as other types of CifB. In addition, more modules were predicted from *w*StriCN CifB proteins. One putative cyclic bacteriocin was predicted downstream of the PDDEXK domains in wStriCN_01615. Both wStriCN_00174 and wStriCN_01406 were much longer than any CifB proteins across Types I-IV and contained the following domains: a TcdA_TcdB_pore domain, a DUF3491 domain of unknown function, a RTX_C domain, an ANK, and a latrotoxin_C domain, respectively (fig. 6*B*). The Ulp1/Proteases domain, which has been experimentally proved to cause CI and exists only in Type I CifB proteins (Beckmann et al. 2017; Lindsey et al. 2018), was also detected in wStriCN_01406. As the longest *CifB*, wStriCN_01406 also encodes one lipoprotein domain, which was similar to Lepidopteran low molecular weight (30 kDa) lipoprotein. It is noteworthy that none of the three *cifA–cifB* gene-pairs are located in predicted WO regions based on the prediction results of PHASTER.

### Biotin and Riboflavin Synthesis Pathways in *w*StriCN

Like most other *Wolbachia* strains, *w*StriCN contained complete pathways for only essential energy-related metabolisms like the tricarboxylic acid cycle, biosynthesis of fatty acid and lipoid acid, and oxidative phosphorylation, which indicate that *w*StriCN has very limited capabilities of biosynthesizing biological macromolecules and metabolic intermediates. As a result, *w*StriCN has to reply on host cells to get nutrients for survival. On the other hand, the *w*StriCN genome has genes for synthesizing the vitamin B members, biotin and riboflavin, which suggests *w*StriCN could contribute essential nutrients that are poor in plant sap to host planthoppers.

## Discussion

The characteristics of the *w*StriCN genome revealed in this study, such as small genome size (compared with other free-living bacteria), low GC content, and incomplete metabolic abilities, indicate that *w*StriCN falls in the range of facultative symbionts (Moran et al. 2008; McCutcheon and Moran 2012; Lo et al. 2016). Previous phylogenetic analyses of *Wolbachia* have assigned *w*StriCN into the supergroup B (Zhang et al. 2013). Our phylogenic trees of both ribosomal proteins and single-copy ortholog proteins are consistent with this classification and show that *w*StriCN is closely related to *w*Di. Our genome-wide ANI analysis confirms the close relationship between *w*StriCN and *w*Di. Therefore, *w*StriCN and *w*Di may have evolved from the same *Wolbachia* ancestor. Although genomes of *w*StriCN and *w*Di are highly collinear and have similar GC content, the genome size of *w*StriCN is almost 50% larger than that of *w*Di and is the largest of all reported supergroup B strains to date. Because the size of a genome is positively correlated with the number of CDSs in bacteria (Lo et al. 2016), *w*StriCN is hypothesized to have more biological functions than other *Wolbachia* strains.

Some arthropod *Wolbachia* strains carry a large number of mobile elements, such as IS and WO (Kent and Bordenstein 2010; Bouchon et al. 2011), which may explain the expansion of *Wolbachia* genomes. Our results show that *Wolbachia* strains have a diverse range of IS genes, ranging from 17 to 217. The distribution pattern of IS genes also varies significantly among different strains. The incomplete draft genome of *w*StriCN has 78 IS genes, more than the medium number in known complete *Wolbachia* genomes. Furthermore, IS genes of *w*StriCN belong to 10 IS families, which is the second highest number in all analyzed 33 *Wolbachia* genomes. The draft genome of *w*Bol1 has 22 IS genes scattered in 11 IS families, which is the highest record in *Wolbachia* so far (Duplouy et al. 2013). IS are simple small transposase-encoding transposable elements that are frequently detected in prokaryotic genomes (Siguier et al. 2006). They can move from one position on a chromosome to a different position on the same or a different one and have important and spectacular effects in shaping and reshuffling bacterial genomes. Although the accumulation of nucleotide substitutions and deletions over time degraded the vast majority of *Wolbachia* IS copies (>70) (Bouchon et al. 2011), the high diversity of IS genes in the *w*StriCN genome showed frequent events of horizontal gene transfer between *w*StriCN and other organisms. The expansion of *w*StriCN genome may be a result of multiple exchanges of DNA fragments mediated by IS elements.

Bacteriophages can carry out lateral gene transfer and shuttle large portions of DNA into recipient genomes (Bordenstein and Wernegreen 2004). Nearly, all sequenced *Wolbachia* harbor prophage WO, except for those acting as obligate mutualistic symbionts (Gavotte et al. 2007; Bordenstein and Bordenstein 2016). The WO in *w*StriCN, with a size of 233.9 kb, are around twice the size of WO in *w*AlbB (Sinha et al. 2019). Large fragments of WO were hypothesized to be responsible for particular phenotypes because they can transfer new functions between phylogenetically distinct strains (Kent and Bordenstein 2010).

Many WO genomes encode putative effectors and toxins, like SpvB, VrlC, and Patatin, that potentially interact with host cells (Kent and Bordenstein 2010). Three copies of Patatin-like phospholipase (PLP) were scattered in the prophage regions of *w*StriCN. PLPs are utilized to facilitate many pathogens’ infection and dissemination (Sitkiewicz et al. 2007). Pathogens and symbionts had significantly higher numbers of PLP-containing genes in their genomes than free‐living bacteria (Banerji and Flieger 2004), which implies that they interact with host cells. For instance, the PLP of intracellular pathogen *Legionella pneumophila* can cleave fatty acids from membrane lipids (Zhu et al. 2013). The release of fatty acids destabilizes the membrane and causes the release of cytochrome C from the mitochondria in the host cells. Cytochrome C activates caspase 3, which in turn activates programed cell death pathways in the host (Zhu et al. 2013). Further studies are needed to determine whether *w*StriCN induces host immune responses in *L. striatellus*.

ANKs can modulate the transcription of host genes by interacting with specific regions of the host chromatin (Al-Khodor et al. 2010). The *w*StriCN genome encodes 113 ANKs, the largest number to date in all reported *Wolbachia* strains. This agrees with previous statistical analysis that showed the number of ANKs is significantly higher in supergroup B than in supergroup A (Lindsey et al. 2016). In addition, ANKs are significantly enriched in the phage regions, which suggests that WO have an important role. The *w*StriCN prophage regions contain 8 genes encoding proteins harboring Zn-binding PAAR, which is a key component of T6SS that can deliver lethal effectors upon direct contact with a target cell (Alteri and Mobley 2016). Bacteria that possess a T6SS have a specific advantage to discriminate, recognize, and kill potential competitors in a population of mixed bacteria.

Apart from T6SS, the *w*StriCN genome encodes secreted proteins and secretion systems that may be key in interacting with host cells. T4SS is prevalent in multiple *Wolbachia* strains (Pichon et al. 2009). It is required for bacterial infection, proliferation, and persistence within hosts. Some pathogenic bacteria use T4SSs to translocate virulence factors into the host cell or to mediate horizontal gene transfer (Grohmann et al. 2018). T1SS bypasses the periplasm and allows the secretion of proteins of diverse sizes as well (Delepelaire 2004). Many ANK-containing effectors of *Wolbachia* relatives, such as *Rickettsia* and *Anaplasma* spp., were found to be translocated by T4SS (Klasson et al. 2008; Al-Khodor et al. 2010; Sinha et al. 2019) and T1SS (Kaur et al. 2012).

*Wolbachia* is well known for inducing CI in many arthropods. Recent genetic studies have shown that the CifA and CifB proteins are mainly responsible for CI in *Drosophila* flies and *Culex* mosquitos (Beckmann et al. 2017; LePage et al. 2017). In addition, CI strength appears to be positively correlated with the number of copies of *cifA* and *cifB* genes in a strain (LePage et al. 2017). For instance, strains with only one copy, such as *w*Mel, have a comparatively weak CI phenotype, whereas those with two or three copies, such as *w*Ri and *w*Ha, cause strong CI (LePage et al. 2017). The facts that *w*StriCN causes strong CI in *L. striatellus* (Noda et al. 2001; Bing et al. 2019) and its genome harbored three sets of *cifA–cifB* gene-pairs strongly support this correlation.

*w*StriCN CifA proteins are phylogenetically and structurally different from other known CifA proteins. The STE domain, which is conserved among all four types of CifA, was missing in all three wStriCN CifA proteins. *w*StriCN CifA proteins also do not have the antioxidant catalase-rel domain that may function in response to reactive oxygen. A genetic analysis suggests that Type I CifA protein alone was able to rescue CI in *Drosophilamelanogaster* (Shropshire et al. 2018). An evolutionary analysis showed that purifying selection is much stronger on the catalase-rel domain than on the Puf family RNA-binding domain and STE domain (Shropshire et al. 2018). So far the catalase-rel domain has been found only in Type I CifA proteins (Lindsey et al. 2018). Although the critical domain that is responsible for the rescue is unclear yet, we speculate that the Puf family RNA-binding domain is important as it is the only domain that exists across all CifA orthologs.

All three *w*StriCN CifB proteins harbor two PDDEXK modules. The Ulp1 ubiquitin proteases module of CifB, which was predicted to be unique and completely conserved in the Type I groups (Lindsey et al. 2018), was also identified in one *w*StriCN CifB (wStriCN_01406). A *Wolbachia* deubiquitylating enzyme (DUB, or ubiquitin proteases) and a PDDEXK (or PD-(D/E)XK) nuclease domain (DUF1703) were predicted to induce embryonic death in CI (Beckmann et al. 2017). All strains that are able to induce or rescue CI have two or more recovered modules, though they do not necessarily have the Ulp1 ubiquitin proteases module (Beckmann et al. 2017; Lindsey et al. 2018). However, whether or not these Cif proteins contribute to CI in *L. striatellus* needs experimental confirmation.

The CifB proteins in *w*StriCN contain many toxin-related domains. wStriCN_01615 encodes one putative cyclic bacteriocin domain, which was reported to have antimicrobial activity (Sawa et al. 2009). Both wStriCN_00174 and wStriCN_01406 encode a TcdA/TcdB pore-forming domain, an RTX C-terminal domain, and a black widow latrotoxin C-terminal domain. The TcdA and TcdB are known as toxins that mediate the pathogenicity of *Clostridium difficile*. They primarily disrupt the cytoskeletal structure and the tight junctions of target cells causing cell rounding and ultimately cell death (Di Bella et al. 2016). The RTX toxin superfamily is a group of cytolysins and cytotoxins produced by bacteria. During transport, the C-terminal repeats of the RTX toxin were recognized by the T1SS and transferred first through the channel (Linhartová et al. 2010). Although latrotoxins are the main toxin generally considered to be exclusively found in spiders, homologs of the latrotoxin C-terminal domain have been reported in bacteria such as *Wolbachia*, and *Rickettsiella grylli* (Zhang et al. 2012), which implies that latrotoxin genes were horizontally transferred from spiders to their bacterial endosymbiont. The latrotoxin C-terminal domain was shown as the most prevalent eukaryoticlike domain in WO (Bordenstein and Bordenstein 2016), indicating that phage might be facilitating its transfer. However, the *w*StriCN Cif genes are distributed out of prophage regions, implying that their evolution is complicated. Further studies are needed to determine whether those toxin-coding regions are active and play roles in interacting with other prokaryotic competitors or eukaryotic hosts.

*Wolbachia* can significantly enhance the fecundity of *L. striatellus*. *Wolbachia*-infected *L. striatellus* females laid 30% more eggs than uninfected females (Guo et al. 2018). Our genomic analysis showed that *w*StriCN contained complete pathways for biological synthesis of biotin and riboflavin, whose concentrations are relatively low in plant sap (Douglas 2017). In contrast to essential amino acids, vitamin Bs were thought to be unimportant nutrition factors for plant sap feeders. However, recent studies in aphids and red cotton bugs showed vitamin Bs contribute to host survival and development as well (Salem et al. 2014; Meseguer et al. 2017). Besides, our recent experimental study stressed the importance of *w*StriCN-provided biotin and riboflavin in *L. striatellus* (Ju et al. 2019).

## Conclusions

The genome of *w*StriCN displays hallmarks of an insect symbiont, including a low GC content and reduced genome size compared with free-living bacteria. On the other hand, *w*StriCN has one of the largest *Wolbachia* genomes with a large number of mobile elements, which have considerable effects on *Wolbachia* genome evolution and gene content*.* Although the Cif proteins in wStriCN are phylogenetically and structurally distinct from all known Cif types, they contain the catalytic domains that correlate with the phenotype of CI and may explain the strong CI phenotype *w*StriCN induces in *L. striatellus*. The genome retains pathways for synthesizing biotin and riboflavin, which helps to explain how *w*StriCN might benefit its host, which feeds on low-nutrient plant sap. Altogether, the *w*StriCN genome is a resource that will provide further insight into the phylogeny of *Wolbachia* and enable further biochemical, molecular, and genetic analyses of *w*StriCN and related symbionts. The genome will also provide clues to the interactions between *Wolbachia* and its host that may lead to advances in pest and disease control.

## Supplementary Material

Supplementary data are available at *Genome Biology and Evolution* online.

## Supplementary Material

evaa006_Supplementary_Data

## Notes

**Data deposition:** The draft genome sequences of *w*StriCN data have been deposited at NCBI GenBank database under the accession MUIX00000000.
