# Supplementary material for: Genomic Analysis of Wolbachia from Laodelphax striatellus (Delphacidae, Hemiptera) Reveals Insights into Its “Jekyll and Hyde” Mode of Infection Pattern
Source: Genome Biol Evol. 2020 Jan 20;12(2):3818–31. doi: 10.1093/gbe/evaa006 (PMC7046167; doi:10.1093/gbe/evaa006)
Supplement: evaa006_Supplementary_Data [file evaa006_supplementary_data.pdf]

Supplementary information

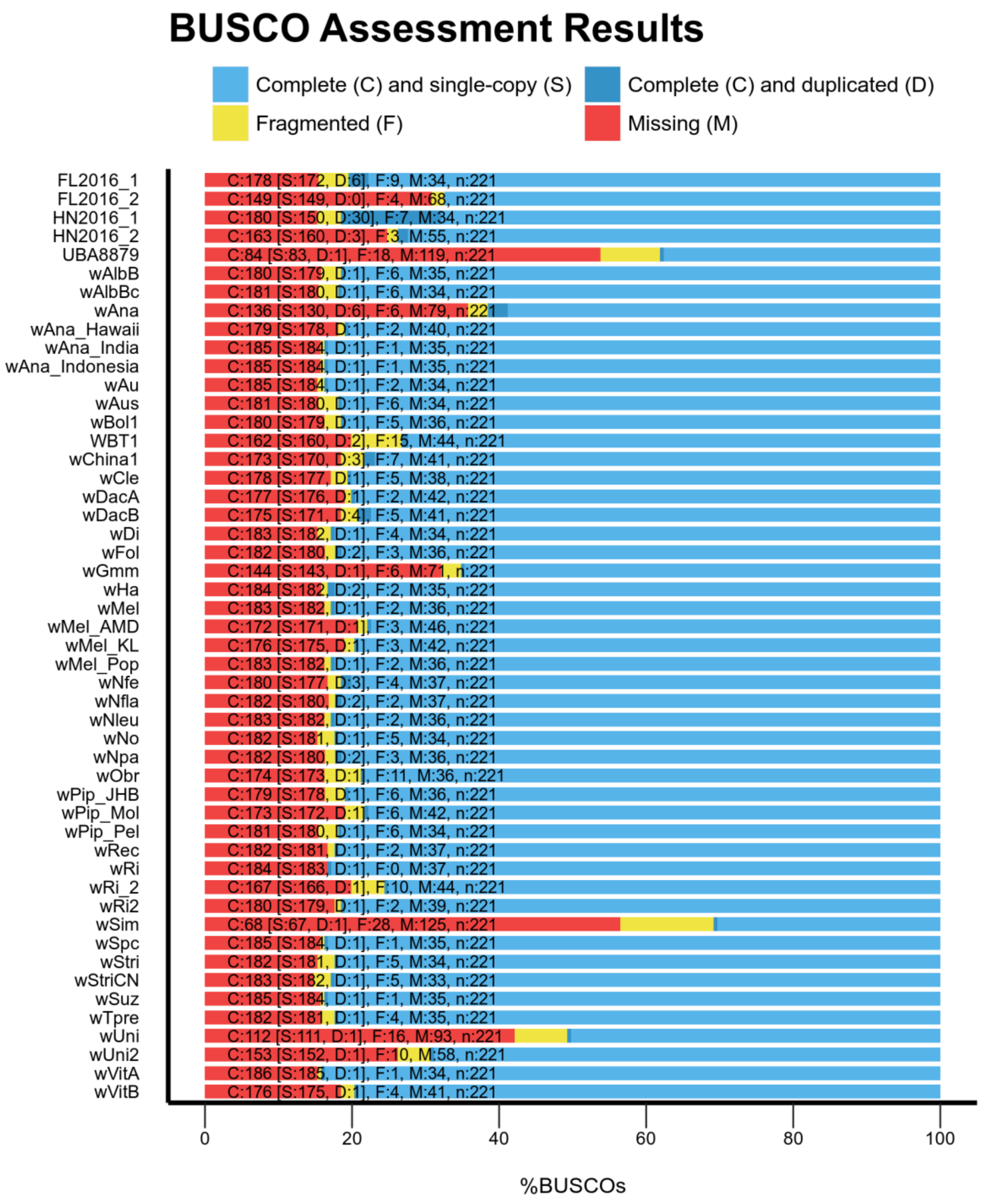

Fig. S1 BUSCO (benchmarking universal single-copy orthologs) assessment result of *Wolbachia* genomes. n, number of genes used.

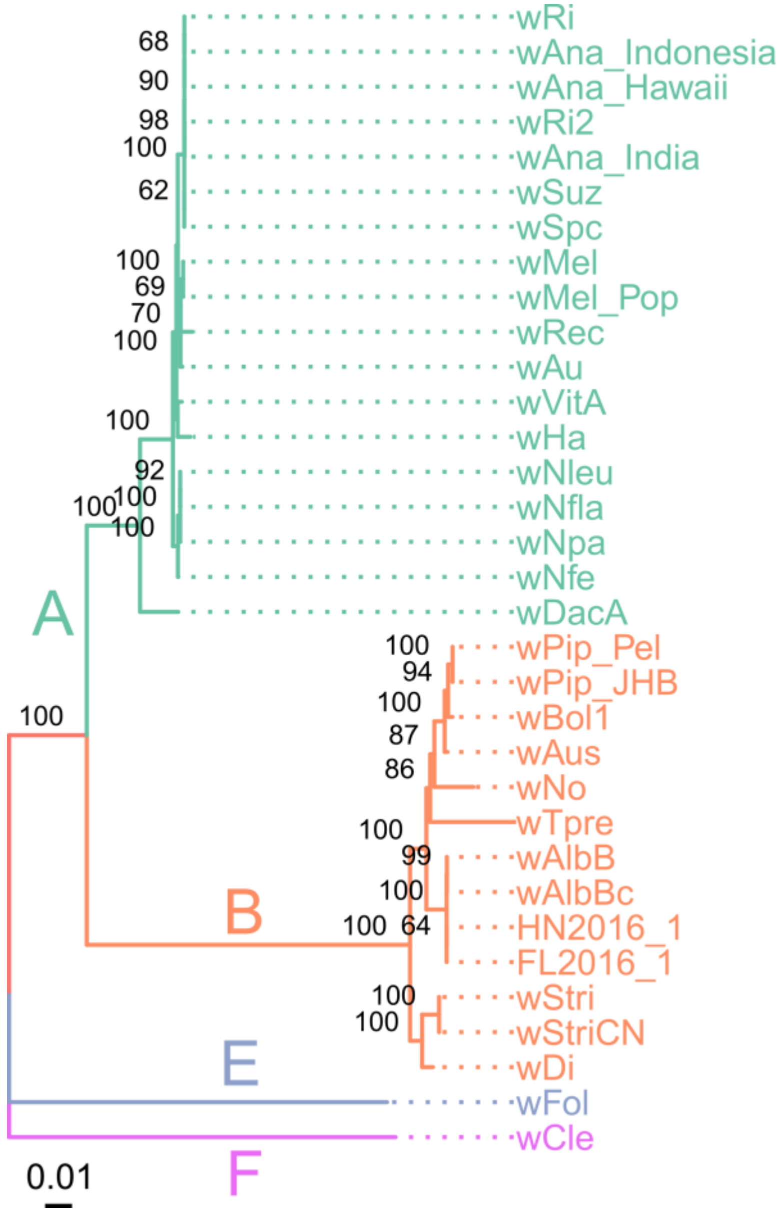

Fig. S2 Phylogenetic relationship of *Wolbachia*.

The Maximum Likelihood (ML) tree was calculated with a concatenated protein sequences of 52 *Wolbachia* ribosomsal genes (7368 amino acids) using a HIVw+F+G4substitution model. *Wolbachia* supergroups are colour coded as shown on the branch.Bootstrap values are indicated at the respective node (only values > 50% are shown). The scale bar represents the average number of substitutions per site.

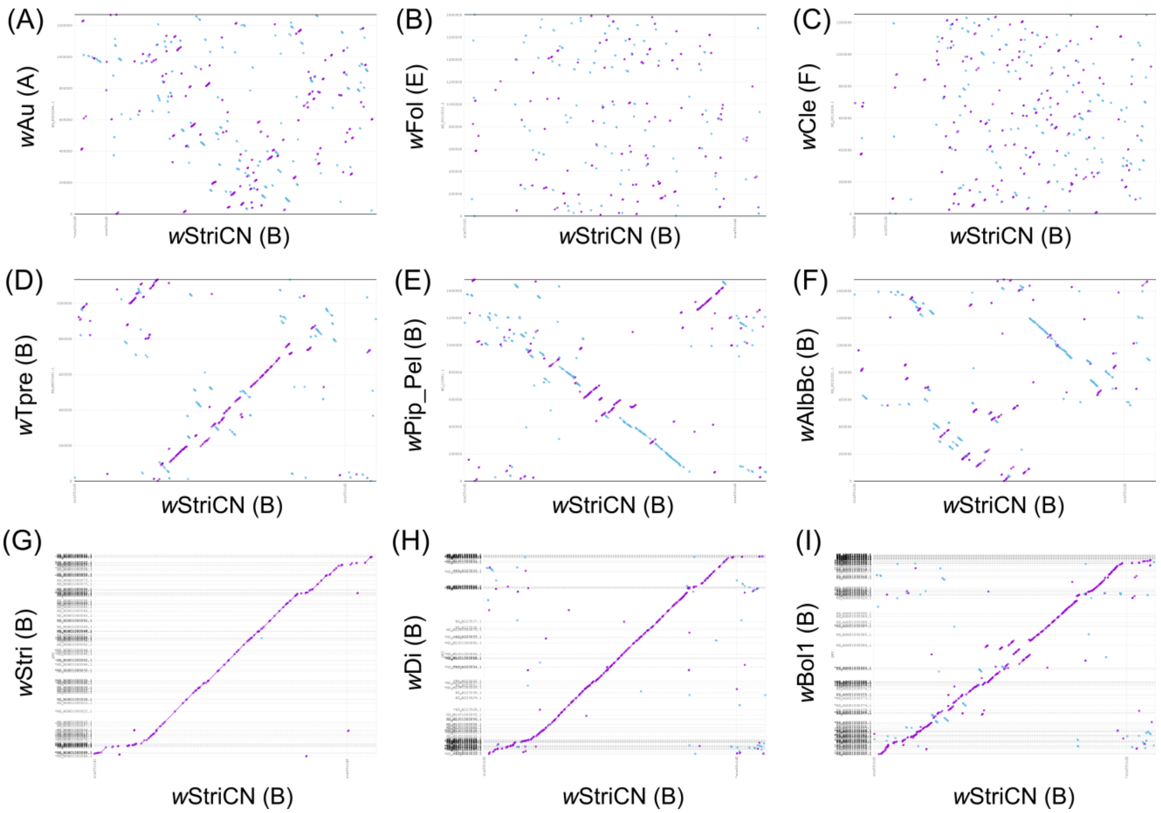

Fig. S3 Mummer plots showing differences in syntenic conversation between *w*StriCN and other *Wolbachia* genomes.

Dots and lines represent unique genomic sequence matches, purple for a forward match and blue for a reverse match (invasions). Numbers along the axes represent genome coordinates. The name and *Wolbachia* supergroup of genomes are shown on axes. wStri, wDi and wBol1 are not complete and contain many scaffolds.

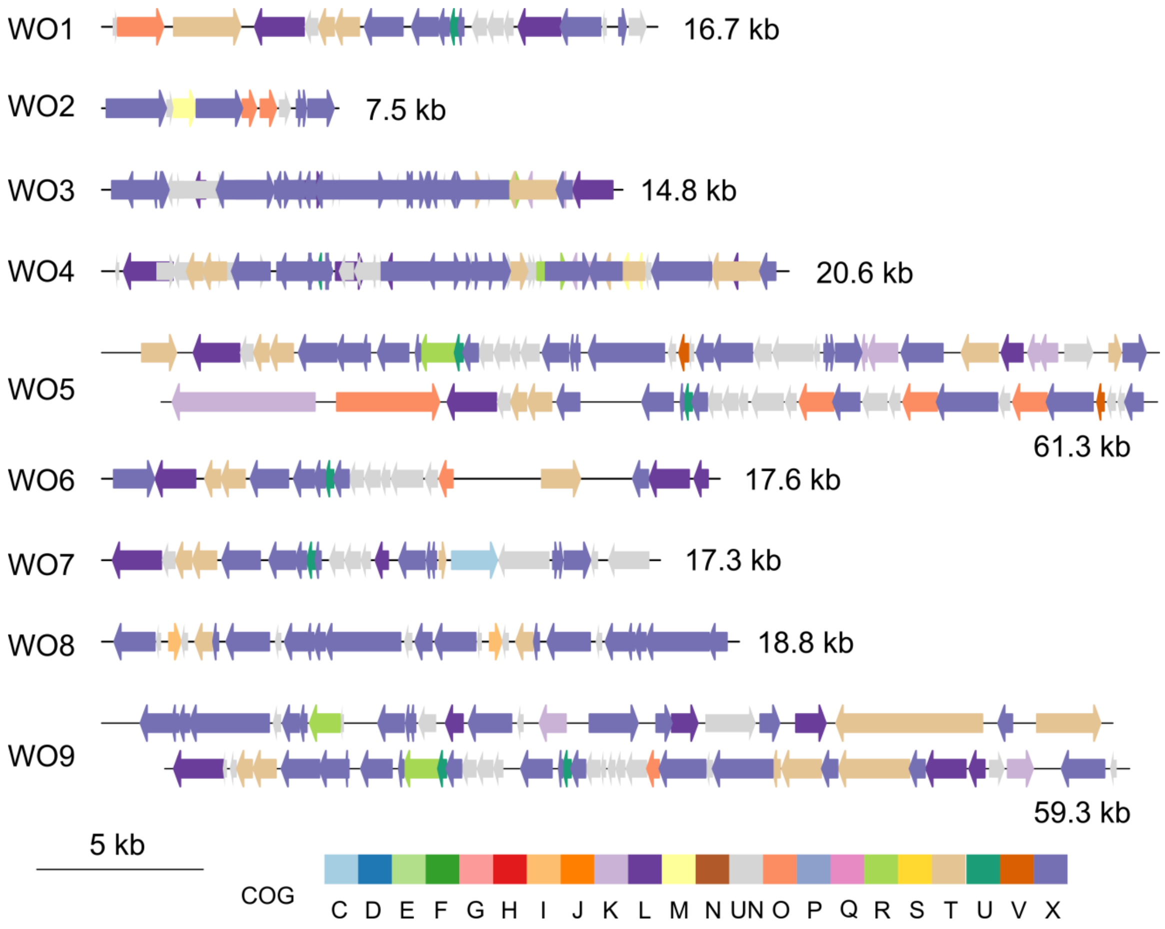

Fig. S4. Detailed graphic view of phage regions in *w*StriCN.

Arrow blocks depict protein coding genes. Gens are color-coded with COG categories. Abbreviations of COG categories are C: Energy production and conversion; D: Cell cycle control, cell division, chromosome partitioning; E: Amino acid transport and metabolism; F: Nucleotide transport and metabolism; G: Carbohydrate transport and metabolism; H: Coenzyme transport and metabolism; I: Lipid transport and metabolism; J: Translation, ribosomal structure and biogenesis; K: Transcription; L: Replication, recombination and repair; M: Cell wall/membrane/envelope biogenesis; N: Cell motility; O: Posttranslational modification, protein turnover, chaperones; P: Inorganic ion transport and metabolism; Q: Secondary metabolites biosynthesis, transport and catabolism; R: General function prediction only; S: Function unknown; T: Signal transduction mechanisms; U: Intracellular trafficking, secretion, and vesicular transport; V: Defense mechanisms; X: Mobilome: prophages, transposons. UN represents genes that have no annotation in COG database.

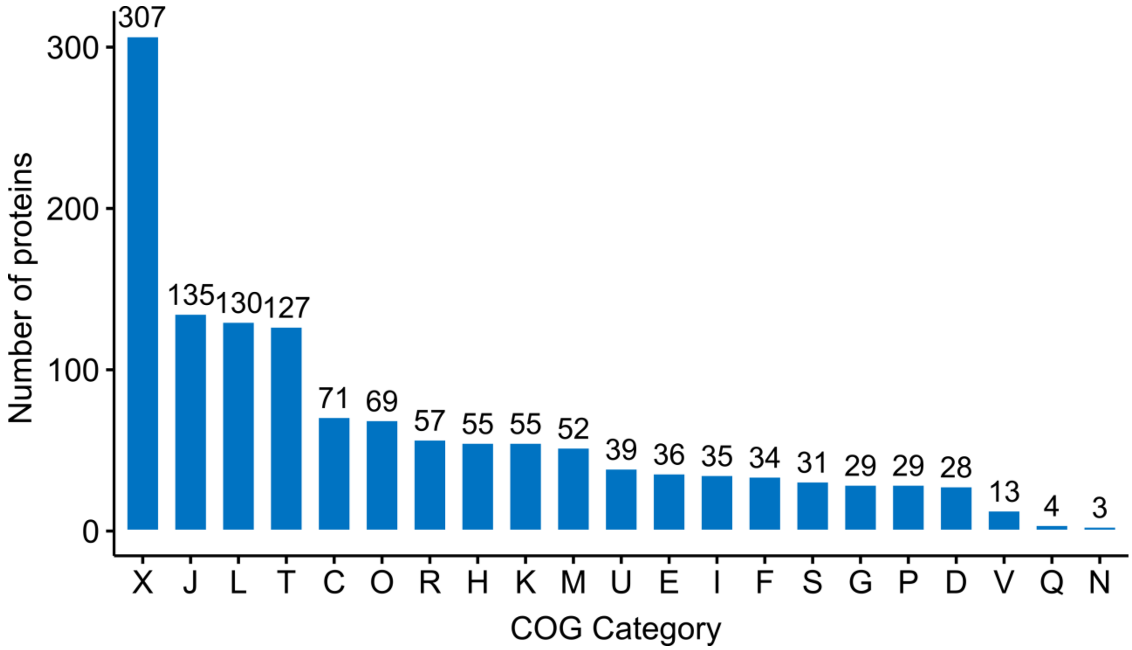

Fig. S5 COG category of *Wolbachia* single-copy ortholog genes

Bar chart showing number of single-copy ortholog proteins in COG categories. Abbreviations of COG categories are described above.

Table S1 Genomic libraries included in the wStriCN genome assembly and their respective assembled sequence coverage levels

Library Type	Average insert size (bp)	Read number (Million)	Read length (Mb)	Assembled sequence coverage (×)	Percentage of *Wolbachia* reads (×)
500 bp (PE)	493	2.33	210.00	117.56	96.65
500 bp PCR-free (PE)	458	2.34	211.00	118.12	94.28
6 kb (MP)	6,740	5.09	457.67	256.2	71.51
454 sequencing		0.65	385.34	215.71	83.84
Total		10.41	1264.01	707.58	-

Note: PE: Pair-End sequence library; MP: Mate-Pair end sequence library.

Table S2 Available *Wolbachia* genomes used in phylogenetic and comparative analyses

Strain	Host_genus	Host_species	Supergroup	Genome	Size (Mb)	GC %	Gene	Protein	Coding density %	BUSCO percentage	RefSeq accession
FL2016_2	*Aedes*	*albopictus*	A	Draft	1.05	35.2	986	832	84.38	67.42	GCF_002379155.1
HN2016_2	*Aedes*	*albopictus*	A	Draft	1.32	38.3	1268	1087	85.73	73.76	GCF_002379175.1
wAna	*Drosophila*	*ananassae*	A	Draft	1.44	35.7	1421	965	67.91	61.54	GCF_000167475.1
wAna_Hawaii	*Drosophila*	*ananassae*	A	Draft	1.22	35.1	1204	1040	86.38	81	GCA_003671405.1
wAna_India	*Drosophila*	*ananassae*	A	Draft	1.24	35.1	1210	1068	88.26	83.71	GCF_003671365.1
wAna_Indonesia	*Drosophila*	*ananassae*	A	Draft	1.22	35.1	1184	1054	89.02	83.71	GCA_003671375.1
wAu	*Drosophila*	*simulans*	A	Complete	1.27	35.2	1265	1099	86.88	83.71	GCF_000953315.1
wDacA	*Dactylopius*	*coccus*	A	Draft	1.17	35.1	1220	984	80.66	80.09	GCF_001648025.1
wGmm	*Glossina*	*morsitans*	A	Draft	1.02	35.1	837	800	95.58	65.16	GCF_000689175.1
wHa	*Drosophila*	*simulans*	A	Complete	1.30	35.1	1263	1126	89.15	83.26	GCF_000376605.1
wMel	*Drosophila*	*melanogaster*	A	Complete	1.27	35.2	1270	1100	86.61	82.81	GCF_000008025.1
wMel_AMD	*Drosophila*	*melanogaster*	A	Draft	1.12	35.1	1100	943	85.73	77.83	GCA_002907445.1
wMel_KL	*Drosophila*	*melanogaster*	A	Draft	1.11	35.1	1095	940	85.84	79.64	GCF_002907525.1
wMel_Pop	*Drosophila*	*melanogaster*	A	Draft	1.24	35.6	1165	1029	88.33	82.81	GCF_000475015.1
wNfe	*Nomada*	*ferruginata*	A	Draft	1.34	35.2	1364	1078	79.03	81.45	GCF_001675785.1
wNfla	*Nomada*	*flava*	A	Draft	1.33	35.2	1354	1090	80.50	82.35	GCF_001675695.1
wNleu	*Nomada*	*leucophthalma*	A	Draft	1.37	35.2	1382	1123	81.26	82.81	GCF_001675715.1
wNpa	*Nomada*	*panzeri*	A	Draft	1.34	35.2	1372	1102	80.32	82.35	GCF_001675775.1
wRec	*Drosophila*	*recens*	A	Draft	1.13	35.2	1132	965	85.25	82.35	GCF_000742435.1
wRi	*Drosophila*	*simulans*	A	Complete	1.45	35.2	1403	1254	89.38	83.26	GCF_000022285.1
wRi_2	*Drosophila*	*simulans*	A	Draft	1.07	35.0	1132	1063	93.90	75.57	GCA_002907425.1
wRi2	*Drosophila*	*simulans*	A	Draft	1.12	35.0	1092	965	88.37	81.45	GCA_002907405.1
wSim	*Drosophila*	*simulans*	A	Draft	1.06	35.4	761	760	99.87	30.77	GCA_000167495.1
wSpc	*Drosophila*	*subpulchrella*	A	Draft	1.42	35.7	1438	1239	86.16	83.71	GCF_002300525.1
wSuz	*Drosophila*	*suzukii*	A	Draft	1.42	35.2	1413	1225	86.69	83.71	GCF_000333795.1
wUni	*Muscidifurax*	*uniraptor*	A	Draft	0.87	35.1	912	827	90.68	50.68	GCF_000174095.1

wVitA	*Nasonia*	*vitripennis*	A	Draft	1.21	34.9	1150	1042	90.61	84.16	GCA_001983615.1
wWil	*Drosophila*	*willistoni*	A	Draft	0.86	35.0			NA	NA	GCA_000153585.1
FL2016_1	*Aedes*	*albopictus*	B	Draft	1.18	33.9	1148	976	85.02	80.54	GCF_002379145.1
HN2016_1	*Aedes*	*albopictus*	B	Draft	1.52	33.9	1504	1240	82.45	81.45	GCF_002374845.1
wAlbB	*Aedes*	*albopictus*	B	Draft	1.16	33.8	1110	955	86.04	81.45	GCF_000242415.2
wAlbBc	Aedes	albopictus	B	Complete	1.48	34.4	1429	1197	83.76	81.9	GCF_004171285.1
wAus	*Plutella*	*australiana*	B	Draft	1.16	34.0	1125	982	87.29	81.9	GCA_002318985.1
wBol1	*Hypolimnas*	*bolina*	B	Draft	1.38	33.9	1293	1139	88.09	81.45	GCF_000333775.1
WBT1	*Bemisia*	*tabaci*	B	Draft	1.25	34.0	1302	979	75.19	73.3	GCF_900097055.1
wChina1	*Bemisia*	*tabaci*	B	Complete	1.31	35.1	1217	965	79.29	78.28	GCF_003999585.1
wCoc1	*Dactylopius*	*coccus*	B	Draft	1.12	34.3	NA	NA	NA	NA	GCA_000832695.1
wDacB	*Dactylopius*	*coccus*	B	Draft	1.50	34.0	1530	1041	68.04	79.19	GCF_001648015.1
wDi	*Diaphornia*	*citri*	B	Draft	0.75	34.0	688	617	89.68	82.81	GCF_000331595.1
wNo	*Drosophila*	*simulans*	B	Complete	1.30	34.0	1231	1065	86.52	82.35	GCF_000376585.1
wObr	*Operophtera*	*brumata*	B	Draft	1.12	33.8	1118	952	85.15	78.73	GCF_001266585.1
wPip_JHB	*Culex*	*quinquefasciatus*	B	Draft	1.54	34.2	1484	1286	86.66	81	GCF_000156735.1
wPip_Mol	*Culex*	*molestus*	B	Draft	1.44	31.7	1268	1231	97.08	78.28	GCF_000723225.2
wPip_Pel	*Culex*	*quinquefasciatus*	B	Complete	1.48	34.2	1402	1257	89.66	81.9	GCF_000073005.1
wStri	*Laodelphax*	*striatellus*	B	Draft	1.23	33.8	1154	1011	87.61	82.35	GCF_001637495.1
**wStriCN**	***Laodelphax***	***striatellus***	**B**	**Draft**	**1.78**	**33.7**	**1882**	**1747**	**92.83**	**82.81**	
wTpre	*Trichogramma*	*pretiosum*	B	Complete	1.13	33.9	1106	827	74.77	82.35	GCF_001439985.1
wVitB	*Nasonia*	*vitripennis*	B	Draft	0.23	34.0	250	150	60.00	79.64	GCF_000204545.1
wFol	*Folsomia*	*candida*	E	Complete	1.80	34.4	1649	1509	91.51	82.35	GCF_001931755.2
wCle	*Cimex*	*lectularius*	F	Complete	1.25	36.3	1246	981	78.73	80.54	GCF_000829315.1
UBA8879				Draft	0.67	33.4	672	627	93.30	38.01	GCF_003516275.1
wUni2	*Muscidifurax*	*uniraptor*		Draft	1.05	35.2	1050	856	81.52	69.23	GCA_001983635.1

Note: Genomic assembly of wWil and wCoc1 are not available for analysis (NA).

Table S3 Genome-wide average nucleotide identity (ANI) between wStriCN and other *Wolbachia*

Wolbachia strain	OrthoANI (%)
FL2016_1	95.16
HN2016_1	95.01
wAlbB	95.16
wAlbBc	95.19
wAna_Hawaii	86.28
wAna_India	86.13
wAna_Indonesia	86.15
wAu	86.39
wAus	95.53
wBol1	94.85
wCle	83.56
wDacA	86.16
wFol	80.67
wHa	86.34
wMel	86.33
wMel_Pop	86.34
wNfe	86.41
wNfla	86.50
wNleu	86.51
wNo	94.02
wNpa	86.39
wPip_JHB	94.62
wPip_Pel	94.78
wRec	86.08
wRi	86.39
wRi2	86.16
wSpc	86.13
wSuz	86.45
wTpre	93.56
wVitA	87.15
wDi	96.17
wStri	99.75

Table S4 Summary of IS families in *Wolbachia*

Species	IS110	IS1380	IS200/ IS605	IS256	IS3	IS4	IS481	IS5	IS6	IS630	IS66	IS982	ISL3	Sum
FL2016_1	1	0	0	0	1	1	0	0	0	0	0	1	1	5
HN2016_1	1	0	0	0	1	1	0	0	0	0	0	0	1	4
wAlbB	0	0	0	0	1	1	0	0	0	0	0	0	1	3
wAlbBc	1	0	0	0	9	1	68	0	0	0	18	94	1	192
wAna_Hawaii	5	1	0	0	0	6	1	2	0	4	2	1	2	24
wAna_India	6	1	0	0	0	5	1	2	0	5	4	1	2	27
wAna_Indonesia	7	1	0	0	0	5	2	2	0	5	4	1	2	29
wAu	9	7	0	0	0	13	1	24	0	3	1	0	2	60
wAus	0	1	0	5	0	0	0	0	1	2	1	0	1	11
wBol1	3	1	0	4	1	0	3	1	2	3	1	2	1	22
wCle	4	0	0	0	0	1	0	208	0	0	0	0	4	217
wDacA	3	1	0	2	0	2	1	19	0	29	0	0	1	58
wDi	6	0	0	1	0	0	0	4	0	0	0	0	1	12
wFol	25	0	0	2	0	20	0	13	0	0	0	3	2	65
wHa	11	0	0	0	0	19	3	2	0	3	4	1	2	45
wMel	8	6	0	1	0	16	1	28	0	3	1	0	2	66
wMel_Pop	6	1	0	1	0	7	1	4	0	1	0	0	2	23
wNfe	8	0	0	2	0	10	2	7	0	4	1	2	3	39
wNfla	7	0	0	2	0	10	2	8	0	1	1	2	3	36
wNleu	7	0	0	2	0	10	3	7	0	2	2	2	3	38
wNo	19	6	0	2	0	2	0	2	0	0	0	0	1	32
wNpa	8	0	0	2	0	9	2	8	0	3	1	2	2	37
wPip_JHB	9	3	0	35	0	0	0	2	8	15	1	53	1	127
wPip_Pel	3	3	0	39	0	0	0	2	7	11	1	52	1	119
wRec	7	1	0	0	0	11	1	14	0	1	1	0	2	38
wRi	27	9	0	0	0	9	7	43	0	13	37	1	2	148
wRi2	5	0	0	0	0	2	1	2	0	3	2	1	1	17
wSpc	41	4	0	0	0	14	4	37	0	12	12	1	2	127
wStri	2	0	0	1	0	0	0	3	1	2	0	0	2	11
wStriCN	18	9	8	2	21	0	0	8	1	2	0	6	3	78
wSuz	25	9	0	0	0	8	2	20	0	11	22	1	2	100
wTpre	1	0	0	0	0	13	2	0	0	0	0	0	1	17
wVitA	2	1	0	0	0	3	1	2	0	0	1	1	2	13
Sum	285	65	8	103	34	199	109	474	20	138	118	228	59	

Table S5 PHASTER predicted prophages from wStriCN genome

WO	Seqname	start	end	Length (Kb)	Completeness	Specific Keyword
WO1	scaffold1	66	16787	16.7	incomplete	integrase,tail
WO2	scaffold1	83049	90581	7.5	incomplete	terminase,portal,capsid,transposase
WO3	scaffold1	94364	109251	14.8	intact	transposase,tail,portal,terminase
WO4	scaffold1	166869	187526	20.6	incomplete	integrase,tail
WO5	scaffold1	233933	295249	61.3	intact	integrase,tail,transposase,plate,capsid,portal,terminase
WO6	scaffold1	1288808	1306494	17.6	incomplete	integrase,tail,capsid
WO7	scaffold1	1368508	1385819	17.3	incomplete	integrase,tail,transposase
WO8	scaffold1	1581451	1600254	18.8	intact	tail
WO9	scaffold2	5544	64915	59.3	intact	tail,transposase,integrase,plate,head,portal,terminase

Table S6 Summary of COG categories in *Wolbachia*

Species	C	D	E	F	G	H	I	J	K	L	M	N	O	P	Q	R	S	T	U	V	X	Sum
FL2016_1	71	28	38	34	28	50	31	133	29	72	52	4	59	27	5	39	35	63	31	6	29	864
HN2016_1	100	34	45	42	31	58	41	173	40	95	64	7	76	32	3	45	37	88	36	9	36	1092
wAlbB	70	27	35	34	26	45	30	131	30	63	45	4	60	25	3	35	28	60	31	8	21	811
wAlbBc	72	30	35	34	32	49	30	136	32	68	51	4	61	26	4	56	30	70	31	9	280	1140
wAna_Hawaii	71	27	37	35	28	48	30	136	32	74	48	3	63	26	4	44	33	51	34	10	75	909
wAna_India	72	28	38	34	28	49	29	134	31	76	48	3	64	26	4	49	33	52	32	10	80	920
wAna_Indonesia	72	29	38	34	28	49	29	132	31	77	49	3	63	26	4	49	33	52	32	10	69	909
wAu	71	28	39	35	31	49	29	134	33	93	47	3	61	26	4	46	33	53	32	17	169	1033
wAus	70	26	35	34	26	49	32	133	21	62	45	3	63	26	3	41	29	64	32	7	42	843
wBol1	69	30	38	34	28	52	34	134	32	86	52	4	68	26	4	49	32	88	34	8	78	980
wCle	72	29	36	36	32	56	29	133	24	87	47	2	60	29	4	49	32	58	30	14	259	1118
wDacA	63	28	35	34	25	46	30	134	29	75	40	3	59	26	3	45	33	46	32	9	108	903
wDi	70	27	34	34	25	49	32	134	31	83	47	3	64	26	3	37	33	76	33	7	42	890
wFol	66	25	34	34	26	46	32	133	36	129	53	3	63	26	4	66	37	111	35	16	191	1166
wHa	71	29	38	36	27	50	28	135	27	87	50	3	58	26	4	50	34	51	34	12	155	1005
wMel	71	30	40	34	30	49	29	135	32	91	47	3	59	26	4	44	35	48	32	17	176	1032
wMel_Pop	71	30	40	34	29	49	29	134	32	83	47	3	59	27	4	42	34	46	32	16	77	918
wNfe	71	26	41	37	30	51	30	137	39	89	48	3	61	26	3	53	33	64	32	14	114	1002
wNfla	72	26	41	36	29	55	30	135	42	86	48	3	60	26	4	56	34	61	32	14	115	1005
wNleu	72	26	41	36	30	55	30	136	42	92	48	3	61	26	4	56	34	65	34	15	123	1029
wNo	70	29	38	37	27	50	32	134	28	84	47	3	60	26	4	48	30	82	32	11	93	965
wNpa	72	26	45	35	31	53	30	137	39	86	49	4	61	25	4	52	34	60	34	13	115	1005
wPip_JHB	70	30	35	34	26	50	33	132	38	113	45	4	70	26	3	57	34	86	35	10	231	1162
wPip_Pel	70	29	35	35	26	50	33	134	34	92	45	3	71	26	3	51	33	86	36	8	213	1113
wRec	72	30	38	34	26	46	29	135	25	73	43	3	57	27	3	42	32	44	31	16	103	909
wRi	72	29	40	34	31	50	29	133	35	94	52	3	64	26	5	83	34	57	34	12	223	1140
wRi2	69	22	37	32	27	47	28	130	29	68	46	3	56	24	3	45	30	51	32	9	48	836
wSpc	72	27	39	34	28	50	30	136	31	86	48	3	63	27	4	56	33	46	33	10	234	1090
wStri	71	28	36	34	27	49	32	134	28	77	48	3	59	28	4	39	30	72	31	12	37	879
wStriCN	71	28	36	34	29	55	35	135	55	130	52	3	69	29	4	57	31	127	39	13	307	1339
wSuz	72	27	40	34	30	50	29	134	35	95	52	3	63	26	5	66	34	51	34	12	184	1076
wTpre	70	32	34	34	33	51	34	135	35	70	53	4	61	30	3	44	31	90	30	11	54	939
wVitA	78	26	41	34	27	49	28	132	30	84	48	3	63	27	4	41	35	53	33	11	62	909

Table S7. wStriCN specific genes identified form ortholog analysis

wStriCN gene	Gene length	Percent identity	evalue	bitscore	NCBI blast hit	blast hit organism[a]
wStriCN_00132	321	89.4	8.93E-108	401	CP015510.2	*Folsomia candida* strain Berlin, complete genome
wStriCN_00210	652	97.1	0	1099	AM999887.1	*Culex quinquefasciatus* Pel strain wPip complete genome
wStriCN_00495	182	93.4	3.40E-67	267	CP034335.1	*Drosophila mauritiana* strain wMau chromosome, complete genome
wStriCN_00954	207	95.2	1.54E-85	327	CP016430.1	*Bemisia tabaci* strain China 1 genome
wStriCN_01336	106	97.2	1.64E-40	178	JX987265.2	*Sogatella furcifera* isolate SfuIS1829(HP) insertion sequence transposase
wStriCN_01369	321	89.4	8.93E-108	401	CP015510.2	*Folsomia candida* strain Berlin, complete genome
wStriCN_01613	329	86.6	2.54E-93	353	CP034335.1	*Drosophila mauritiana* strain wMau chromosome, complete genome
wStriCN_01750	230	96.5	4.19E-101	379	CP015510.2	*Folsomia candida* strain Berlin, complete genome
wStriCN_01832	368	100.0	0	680	MH210682.1	*Sogatella furcifera* strain wSfur adenosylmethionine-8-amino-7-oxononan
wStriCN_01855	372	99.2	0	671	AE017196.1	*Drosophila melanogaster*, complete genome

[a] blast hit organisms shown are hosts of *Wolbachia* endosymbiont.
